# Validation of the “My Headache Checker” that includes osmophobia in the diagnosis of migraine

**DOI:** 10.1002/jgf2.368

**Published:** 2020-09-03

**Authors:** Masahide Matsushita, Kaori Matsumoto, Satoko Kitamura, Naoki Komatsu, Hiromi Seo, Seisho Takeuchi

**Affiliations:** ^1^ Kochi General Rehabilitation Hospital Kochi Japan; ^2^ Department of General Medicine Kochi Medical School Hospital Nankoku Japan

**Keywords:** “My Headache Checker”, migraine, screener, the ID‐Migraine™

## Abstract

**Background:**

Migraine is a common headache disorder, with a 1 year prevalence rate of 6.0 %. However, less than 10% of patients with migraine receive medication in hospital. “My Headache Checker,” a brief and self‐administered migraine screening tool, which includes osmophobia in addition to the ID‐Migraine™ three‐item subset, was developed. The objective of this study was to analyze the applicability of “My Headache Checker” in Japanese patients.

**Methods:**

A total of 238 patients visiting the outpatient department were enrolled in the study. The patients’ chief complaint was not headache. “My Headache Checker” was administered to the patients. Subsequently, they were evaluated by a generalist for the diagnosis of headache. The clinical diagnosis of headache was determined based on the International Classification of Headache Disorders Ⅲ.

**Results:**

Twenty (8.4%) patients satisfied the criteria for the diagnosis of migraine. Sensitivity, specificity, positive predictive value, and negative predictive value of “My Headache Checker” were 0.90, 0.83, 0.69, and 0.95, respectively. Sensitivity, specificity, positive predictive value, and negative predictive value of the ID‐Migraine™ were 0.90, 0.85, 0.72, and 0.95, respectively.

**Conclusion:**

The majority of migraine patients are missed in busy outpatient departments. Our results suggest that “My Headache Checker” is a useful tool in diagnosing unrecognized migraine patients. However, the addition of osmophobia did not contribute to improve the screening power of the ID‐Migraine™.

## 1 INTRODUCTION

Headache is a major worldwide health problem, and the second most common type is migraine. Migraine is a common headache disorder, with a 1 year prevalence rate of 6.0 %. Migraine mainly affects adults during the most productive years of life. In fact, it has been shown that 20% of people affected by a migraine have difficulties in working.^1^ Migraine is rated as one of the most disabling disorders by the *World Health Organization*.^2^ Migraine is still underdiagnosed and inappropriately treated. Yet, less than 10% of patients with migraine receive medication in hospital. In many cases, a proper diagnosis and treatment may take years.^3^ The inadequate medical ability to diagnose and treat migraine could cause frequent care visits at output and emergency department.^4^ Migraine increases job absenteeism and leads to reduction in quality of life.^5^ Recently developed triptans are very effective for migraine. Therefore, an accurate diagnosis of migraine is critical to improve these patients’ quality of life. Taking a detailed medical history is time‐consuming but necessary to diagnose migraine correctly. This limits its application particularly in primary care settings. A simple screening tool will result in a rapid recognition of migraine so that appropriate management can be commenced without delay.

The ID‐Migraine™ questionnaire was designed to establish the validity and reliability of a brief, self‐administered migraine screening tool for patients with headache complaints, in a primary care setting.^6^ It has been developed with the aim of assisting physicians to identify migraine patients in the shortest possible time. A high internal consistency of the ID‐Migraine™ test was observed. Questions regarding disability, nausea, and photophobia (sensitivity to light) were the most predictive factors for the diagnosis of migraine, with adjusted odds ratios of 3.3, 3.9, and 3.8, respectively. It can optimize the management of migraine patients with an important saving time.

Migraine symptoms’ prevalence differs depending on the regions and racial groups.^7^ For example, Asian studies have reported a lower prevalence of photophobia compared to Western studies. On the contrary, osmophobia (sensitivity to odor) had a higher prevalence in Asian migraine patients.^8^ “My Headache Checker” was developed in Japan and includes four diagnostic screening questions.^9^ Specifically, it includes osmophobia (sensitivity to odor) in addition to the ID‐Migraine™ three‐item subset (disability, nausea, and photophobia). The objective of this study was to analyze the applicability of “My Headache Checker” in Japanese patients attending the Department of General Medicine for any reasons, as well as to determine the prevalence of migraine and the ratio of hidden migraine.

## 2 METHODS

### 2.1 Study setting and design

We performed a cross‐sectional study conducted at a 605‐bed tertiary care general hospital. All adult patients over 20 years of age capable of communicating, regardless of the reason for consultation, were recruited to the study over a 10 month period. The consecutive output patients of both genders attending the Department of General Medicine, whose chief complaint was not headache, were included in the study. Patients unable to complete questionnaires or to understand the study consent were excluded. Individuals with psychiatric disorder, cognitive deficits, or disorders that could interfere in the oral communication were also excluded. After accepting an informed consent, 238 patients participated in the study.

### 2.2 “My Headache Checker”

“My Headache Checker” was developed by The Japanese Headache Society.^9^ Screening questions were selected according to an epidemiological study performed in Japan. Specifically, the four questions are as follows: disability, nausea, photophobia, and osmophobia. Test diagnosis of migraine required at least two positive responses. In contrast, the three questions of the ID‐Migraine™ are as follows: disability, nausea, and photophobia. Test diagnosis of migraine required at least two positive responses.

### 2.3 Clinical protocol

“My Headache Checker” was administered to the patients. Subsequently, they were evaluated by a generalist for the diagnosis of headache. The clinical diagnosis of headache was determined according to the International Classification of Headache Disorders Ⅲ (ICHD‐Ⅲ).^10^ Both migraine with aura and migraine without aura were included in migraine in this study.

### 2.4 Statistical analysis

Descriptive statistics were performed based on responses to “My Headache Checker.” The test screener’s validity was assessed based on sensitivity, specificity, and positive and negative predictive values. Fisher’s exact probability test was used for the analysis of categorical variables, while the Mann‐Whitney *U* test was used for quantitative variables. The analysis was performed using SPSS version 15.0.

### 2.5 Ethical disclosure

The ethics committee of our institution approved the study protocol.

## 3 RESULTS

A total of 238 patients (males, n = 101) with a mean age of 61.9 ± 16.8 years participated in the study. Of these, 67 patients (28.2%) with a mean age of 54.0 ± 17.9 resulted in having a headache. Reasons for the present visit are summarized in Table 1. Headaches were more frequently found in female (38.7%) than in male (13.9%) patients (*P* < .05). When using the ICHD‐Ⅲ criteria, 20 (8.4%) patients satisfied the criteria for the diagnosis of migraine, while 47 patients were included in the “Other Types of Headaches,” as follows: 45 tension‐type headaches (TTH); 1 cluster headache; and 1 headache associated with head trauma. Migraine was more frequently found in female patients (*P* < .05). We observed that patients with migraine were younger than those with “Other Types of Headaches” (*P* < .01).

**Table 1:** Reasons for the present visit: diseases

Diseases	Number of patients	Percentage
Depression	34	14.3
Hypertension	33	13.9
Cardiovascular diseases	24	10.1
Psychiatric diseases	22	9.2
Endocrine‐metabolic diseases	20	8.4
Gastrointestinal‐hepatic diseases	17	7.1
Malignancies	15	6.3
Muscle‐skeletal diseases	14	5.9
Respiratory diseases	14	5.9
Various signs and symptoms	13	5.5
Infectious diseases	11	4.6
Neurological diseases	7	2.9
Urogynecological diseases	7	2.9
Organs of senses diseases	5	2.1
Dermatologic disease	2	0.8

The characteristics of functional disability, nausea, photophobia, and osmophobia were more frequently observed in the 20 patients in the migraine group than in the 47 patients with “Other Types of Headaches” (*P* < .01) (Table 2). Eighteen of 20 migraine group patients (90%) were diagnosed with migraine by “My Headache Checker.” Importantly, only 8 of 47 (17%) “Other Types of Headaches” were diagnosed with migraine by “My Headache Checker” (*P* < .01). In total, 20 patients satisfied the ICHD‐Ⅲ criteria for the diagnosis of migraine. Conversely, 26 patients were diagnosed with migraine by “My Headache Checker.”

**Table 2:** Answers to “My Headache Checker” in Migraine versus Other Types of Headaches

	Migraine	Other types of headaches	*P*
	(n=20)	(n=47)	
Functional disability	17	16	<.01*
Nausea	13	7	<.01*
Photophobia	15	8	<.01*
Osmophobia	8	6	<.05*
Two or more items positive	18	8	<.01*

Note

Numbers represent patients in each group.

* Fisher’s exact probability test.

We observed that sensitivity, specificity, positive predictive value, and negative predictive value of “My Headache Checker” were 0.90, 0.83, 0.69, and 0.95, respectively (Table 3). On the contrary, sensitivity, specificity, positive predictive value, and negative predictive value of the ID‐Migraine™ were 0.90, 0.85, 0.72, and 0.95, respectively. Taken together, we found that “My Headache Checker” was equal in both sensitivity and negative predictive value, while it was slightly inferior in both specificity and positive predictive value. Both positive and negative likelihood ratios of “My Headache Checker” were 5.29 and 0.12, respectively. Both positive and negative likelihood ratios of the ID‐Migraine™ were 6.0 and 0.12, respectively.

**Table 3:** Comparison of the ID‐Migraine^™^ and “My Headache Checker” in the diagnosis of migraine

	Sensitivity	Specificity	PPV	NPV	PLR	NLR
Functional disability	0.85	0.66	0.52	0.91	2.5	0.23
Nausea	0.65	0.85	0.65	0.85	4.33	0.41
Photophobia	0.75	0.83	0.65	0.89	4.41	0.3
Osmophobia	0.4	0.87	0.57	0.77	3.08	0.69
Two or more items positive by “My Headache Checker”	0.9	0.83	0.69	0.95	5.29	0.12
Two or more items positive by the ID‐Migraine^™^	0.9	0.85	0.72	0.95	6	0.12

Abbreviations: NLR, negative likelihood ratio; NPV, negative predictive value; PLR, positive likelihood ratio; PPV, positive predictive value.

## 4 DISCUSSION

In the present study, using the ICHD‐Ⅲ criteria, 8.4% of the patients satisfied the criteria for migraine’s diagnosis. Interestingly, this prevalence is similar to the 6.0% prevalence rate previously reported in Japan.^1^ Notably, none of the patients enrolled in this study had headache as their chief complaint. An earlier study showed that less than 10% of patients with migraine are treated with medication in hospital.^1^ Our findings suggest that most of the migraine patients are missed in busy outpatient departments. Our findings support the use of “My Headache Checker” as a useful tool for the diagnosis of unrecognized migraine patients.

In 2003, Lipton et al. developed the ID‐Migraine™.^6^ It comprises questions regarding disability, nausea, and photophobia (sensitivity to light), and represents a reliable tool with the sensitivity of 0.81, specificity of 0.75, and positive predictive value of 0.93. Validation studies of the ID‐Migraine™ have been performed in numerous settings, including ophthalmology, ENT, neurology outpatient clinics, and emergency department.^11^, ^12^ However, it is not a widely used tool for migraine diagnosis.

It has been shown that osmophobia is recognized in 25%–48% of patients with migraine.^13^, ^14^ In 2005, The Japanese Headache Society developed “My Headache Checker”.^9^ In addition to the three questions included in the ID‐Migraine™, this screener included osmophobia. The validation study of “My Headache Checker” was performed on 307 patients with headache, in 28 institutions. Sensitivity, specificity, and positive predictive value of “My Headache Checker” were reported to be 0.74, 0.85, and 0.91, respectively.^9^

Of note, in order to identify undiagnosed migraine patients, the screener requires higher sensitivity and negative predictive value. However, both sensitivity and negative predictive value of “My Headache Checker” for migraine were equal to those of the ID‐Migraine™. "My Headache checker" is developed by adding the osmophobia on the ID‐Migraine™, and the threshold is two or more items positive in both diagnostic tools. The expected result is higher sensitivity and lower specificity in "My Headache checker" compared to the ID‐Migraine™. However, osmophobia’s low sensitivity could determine a low sensitivity of “My Headache Checker.” In addition, both specificity and positive predictive value were slightly lower in “My Headache Checker.” Therefore, the addition of osmophobia did not contribute to improve the screening power of the ID‐Migraine™. However, “My Headache Checker” is still useful in judging the necessity to prescribe triptans. The fact that negative predictive value was 0.95 is worthy of note.

We identified several limitations in the present study. First, the clinical diagnosis of headache was done by a generalist, not by a headache specialist. A previous study demonstrated that in the diagnosis of migraine headache, there is a low‐level agreement between lay interviewers and headache experts.^15^ Second, the study protocol’s results were not blind. Specifically, the results of “My Headache Checker” were provided to the physicians prior to the patients’ interview. Such an approach may cause a bias in the diagnosis of migraine, regardless of the fact that the diagnosis was made according to the ICHD‐Ⅲ. Third, many Japanese migraineurs have TTH, too. Therefore, it may be difficult to separate migraine and TTH clearly. Finally, the study was conducted in a university hospital. Therefore, it is unsure whether the results can be generalized to the public.

The majority of migraine patients are missed in busy outpatient departments. The results of the present study suggest that “My Headache Checker” is a useful tool in the diagnosis of unrecognized migraine patients. However, the addition of osmophobia did not contribute to improve the screening power of the ID‐Migraine™.

## CONFLICT OF INTEREST

The authors have stated explicitly that there are no conflicts of interest in connection with this article.
